# Effects of a Vegetarian Diet on the Development of Thyroid Disorders

**DOI:** 10.7759/cureus.71360

**Published:** 2024-10-13

**Authors:** Cristina M Prudencio-Brunello, Regina Mae D Palencia, De-Kee Yangzom, Pooja Boddapati, Arun Nair

**Affiliations:** 1 School of Medicine, St. George's University School of Medicine, True Blue, GRD; 2 Medicine, University of Colombo, Colombo, LKA; 3 Medicine, Mediciti Institue of Medical Sciences (MIMS), Hyderabad, IND; 4 Pediatrics, Saint Peter’s University Hospital, Somerset, USA

**Keywords:** autoimmune hypothyroidism, hyperthyroidism, iodine, non-autoimmune hypothyroidism, vegetarian diet

## Abstract

This study aims to explore the impact of different types of vegetarian diets on thyroid health, particularly focusing on the prevalence of thyroid disorders. Vegetarianism has had increasing popularity and has been particularly prominent in Asia, where a large proportion of the population has adopted a vegetarian diet, whereas in North America, the prevalence of vegetarianism remains very small. Vegetarian and vegan diets have been known to pose a risk of deficiencies in minerals and vitamins including vitamin B12, selenium, vitamin A, iron, and iodine, which are crucial for thyroid health. Studies have shown that deficiencies in the aforementioned minerals and vitamins can exacerbate both hyper- and hypothyroidism. This study reviews global cohort studies and highlights the need for a balanced diet with appropriate supplementation of crucial minerals and vitamins involved in thyroid hormone synthesis and further amplifies the need for continued studies and modifications in recommendations to incorporate vegetarian diets as an important confounding factor in the development of thyroid disease.

## Introduction and background

Vegetarian diet is generally understood as excluding meat from one’s diet, but there are different types of vegetarianism depending on how restrictive the diet is. The worldwide prevalence of vegetarianism is not uniform, and North America is known to have a low prevalence with only 6% of the population adopting vegetarianism, while Asia has the highest prevalence of vegetarians, with India contributing most of it as almost 40% of the population is vegetarian [1]. 

Religion and ethical morality are the major driving forces in adopting vegetarianism as an eating habit. Other important motivations are the potential health benefits and sustainability reasons owing to the harmful impact of the meat production industry on the environment. 

Major cohort studies from North America, the United Kingdom, India, and Southeast Asia have studied the trends of vegetarianism and their health outcomes. Following 15 years of the data and assessing nutritional intake, it was apparent that vegans may be at risk for deficiency of Vitamin B12 and iodine [2]. Iodine deficiency in childhood can prevent children from attaining their full physical potential and intellectual capacity [2]. In this study, we will explore the deficiency of microelements leading to the different types of thyroid disorders.

Plant-based diets have a vast variety depending upon certain food types that are limited in the diet. There are flexitarians who occasionally consume meat and poultry but continue to consume fish, egg, and dairy products. Pescatarians do not consume meat and poultry but consume fish, egg, and dairy products. Then we have the lacto-ovo-vegetarians who do not consume meat, poultry, and fish but consume egg and dairy products, whereas the ovo-vegetarians do not consume meat, poultry, fish, and eggs but consume dairy products. Veganism is when people completely avoid all animal products such as meat, poultry, fish, egg, and dairy products. There is also the group of strict raw food consumers where they exclude all animal products and also heated food items [3].

Thyroid hormones are essential in brain and somatic development in infants and are crucial for metabolic activities in adults. Iodine is essential in thyroid hormone synthesis and function. Iodine can only be available in the food that contains it or to which it is added. Both excessive and insufficient intake of iodine can cause thyroid disorders. Iodine levels can be measured by the median urinary iodine concentration to determine its deficiency or sufficiency. Additionally, if there is a deficiency in other elements such as selenium, iron, and Vitamin A, it can exacerbate the effects of iodine deficiency. The thyroid has more selenium than other organs in our body and is involved in the biosynthesis of thyroid hormones. A deficiency of selenium is known to exacerbate the symptoms of cretinism and autoimmune thyroid disorders [4]. Thyroid disorders can also present as excess thyroid hormones causing hyperthyroidism. The prevalence of hyperthyroidism is approximately 1.3% but increases to 4-5% in older women [5]. The common causes are Graves’ disease, which is an autoimmune disorder, overactive thyroid nodules, and thyroiditis. Graves’ disease is more common in younger women, and toxic nodular goiter is more common in older women [5]. 

The data on ethnic differences is minimal, but hypothyroidism is slightly more common in the Caucasian population compared to other races [6]. Roughly one out of 100 Americans aged 12 years and older have hypothyroidism [6]. An underactive thyroid will cause hypothyroidism, the most common cause globally being iodine deficiency. Hashimoto thyroiditis, which is also an autoimmune disorder, is also a common cause of hypothyroidism. The prevalence of hypothyroidism is estimated to be 4.6% of the population of the United States [7]. The differences in iodine intake affect the prevalence of hypothyroidism, which occurs more frequently both in populations with a relatively high iodine intake and in severely iodine-deficient populations, which may also have an increased risk of other autoimmune conditions [8,9]. Studies examining vegetarians, specifically in Western countries, have shown a slightly reduced risk of interstitial heart disease; however, it is suggested that further research may be required to understand the full extent of how a vegetarian diet may impact many facets of health and disease [10].

Our study aims to review the impact of vegetarianism and its effects on the development of these thyroid disorders.

## Review

Key nutrients in thyroid function

Several nutrients play a key role in thyroid function as highlighted in Table 1. Adequate intake of essential nutrients such as iodine, selenium, zinc, and vitamin B12 is critical to support thyroid health [10,11]. Iodine is necessary for the synthesis of thyroid hormones and can be found in seafood, dairy, and certain grains [3,12,13]. Selenium is a vital trace mineral that helps protect the thyroid gland from oxidative damage and regulates immune function. Good plant-based sources of selenium include Brazil nuts, sunflower seeds, and mushrooms. Adequate selenium intake has been associated with reduced thyroid antibody levels and improved glandular function in autoimmune thyroiditis [12,13]. Zinc, abundant in legumes, nuts, and seeds, is necessary for thyroid hormone production and immune function [12]. Vitamin D also plays a role in overall immune modulation and has been linked to autoimmune thyroid conditions. Although primarily obtained from sunlight, vitamin D can also be found in fortified plant milk and supplements [12]. Iron is crucial for thyroid hormone synthesis and overall immune function. Though the main dietary source is meat, iron-rich plant foods such as lentils, chickpeas, tofu, and fortified cereals may be combined with vitamin C-rich foods to enhance absorption and ensure adequate levels [12]. Lastly, vitamin B12 supports overall thyroid health and energy metabolism. It is sourced from animal products such as meat and eggs but can also be obtained from fortified foods and supplements [12].

**Table 1:** Essential nutrients in thyroid function and their dietary sources.

Nutrient	Role in thyroid function	Foods rich in the nutrient	References
Iodine	Critical component of thyroid hormones (T3 and T4)	Iodized salt, seaweed, seafood, dairy products, certain grains	[12,13]
Selenium	Biosynthesis of selenoproteins (i.e., glutathione reductase) which help protect the thyroid gland from oxidative damage	Brazil nuts, sunflower seeds, mushrooms, whole grains, seafood	[12,13]
Zinc	Immunomodulatory	Meat, shellfish, legumes, nuts, and seeds	[12]
Vitamin D	Helps modulate the immune system and inflammation in the thyroid	Sunlight exposure, fatty fish, fortified foods,	[12]
Iron	Enhances thyroid hormone synthesis	Red meat, poultry, seafood, lentils, beans, chickpeas, tofu, spinach, fortified cereals	[12]
Vitamin B12	Supports overall thyroid health and energy metabolism	Meat, dairy products, eggs, fortified foods/supplements	[12]

Vegetarian diets and hypothyroidism

Potential Benefits for Thyroid Health

Vegetarian diets offer benefits for thyroid health through their high percentage of antioxidants and anti-inflammatory compounds, thus promoting healthy weight management [9]. An abundance of antioxidants such as vitamins C and E, polyphenols, and flavonoids can mitigate oxidative stress and inflammation in the thyroid gland [10]. Obesity is a risk factor for hypothyroidism, and vegetarian diets are often associated with lower body mass index (BMI) and reduced risk of obesity [9]. This weight management benefit may indirectly support thyroid health by reducing the strain on the thyroid gland and improving hormone regulation [9,10].

Potential Risks for Thyroid Health

Conversely, certain aspects of vegetarian diets may pose risks for individuals with thyroid disorders if not carefully managed [3,11]. Some plant foods, particularly cruciferous vegetables (such as broccoli, cauliflower, and Brussels sprouts), soy products, and millet, contain goitrogens substances that may interfere with thyroid hormone production [12]. While cooking can reduce goitrogenic activity, high consumption of raw goitrogenic foods may contribute to thyroid dysfunction in susceptible individuals [12]. Iodine is essential for the synthesis of thyroid hormones [12]. Vegetarians may have varied iodine intake depending on their use of iodized salt and consumption of iodine-rich foods like seaweed. Iodine deficiency has been shown to correlate with the development of hypothyroidism [13]. Both iodine deficiency and excess can exacerbate autoimmune thyroid conditions. Vitamin B12, naturally sourced solely from animal products, is essential for neurological function and red blood cell production [14]. Deficiency in vitamin B12 can indirectly affect thyroid health by contributing to overall metabolic and immune system dysfunction. 

Vegetarian diets and autoimmune thyroid conditions

Potential Benefits for Autoimmune Thyroid Health

Well-balanced vegetarian diets are rich in anti-inflammatory compounds, such as polyphenols, flavonoids, and carotenoids [9]. Chronic inflammation is a hallmark of autoimmune disorders, and the anti-inflammatory nature of vegetarian diets may help mitigate this underlying issue. The high fiber content of vegetarian diets also promotes a healthy gut microbiome [9,14]. Emerging research indicates that balanced gut microflora can positively influence the immune system, potentially reducing the severity of autoimmune responses [9].

Potential Risks for Autoimmune Thyroid Health

Foods with high goitrogenic activity or excessive consumption of some plant foods in raw form could impact thyroid function, particularly in individuals with existing thyroid disorders [15,16]. If not well-planned, vegetarian diets may lack certain nutrients critical for thyroid health, such as vitamin B12 and omega-3 fatty acids [14]. Vitamin B12 and selenium deficiency, common in vegetarians, can exacerbate autoimmune thyroid conditions alongside iodine deficiency, emphasizing the need for rigorous nutritional planning to prevent nutrient-related thyroid conditions [15,17,18]. 

Vegetarian diets and hyperthyroidism

Potential Benefits for Thyroid Health

While there have been studies comparing the relationship between a vegetarian diet and autoimmune diseases, studies on the relationship between the incidence of hyperthyroidism and a vegetarian diet are scarce [19]. Studies have documented a lower incidence of autoimmune diseases in rural populations, potentially attributed to the protective effects of their traditional vegetarian diets [19]. Hyperthyroidism has many causes, amongst which the most common etiology is Graves' disease. A cross-sectional study comparing hyperthyroidism prevalence across various diets, including vegan, lacto-ovo vegetarian, pesco-vegetarian, semi-vegetarian, and omnivore diets, found a lower prevalence of hyperthyroidism among vegans, lacto-ovo vegetarians, and pesco-vegetarians [19]. The greatest risk reduction was observed among vegans. Potential confounding factors, such as BMI, were accounted for, and logistic regression was performed to adjust for multiple variables.

Potential Risks for Thyroid Health

While excess iodine in the diet can cause hyperthyroidism, studies have mainly demonstrated the protective mechanism of a vegetarian diet in developing hyperthyroidism. This is achieved by lowering the insulin-like growth factor levels and through flavonoids that protect against autoimmune processes.

Discussion

When comparing diets, the vegan diet offers high antioxidants and anti-inflammatory compounds, supports weight maintenance, and promotes healthy gut microbiota but poses risks of deficiencies in iodine, vitamin B12, iron, and omega-3s as described in Table 2, which provides a general overview of the advantages and disadvantages of the different types of diets currently present. The ovo-lacto vegetarian diet provides similar benefits with antioxidants and weight maintenance, along with adequate vitamin B12 from dairy and eggs, but risks iodine and zinc deficiencies and iron deficiency due to non-heme iron's lower bioavailability. A vegetarian diet, excluding eggs and dairy, shares benefits like high antioxidants and weight maintenance but has a higher risk of iodine, vitamin B12, and iron deficiencies. The pescatarian diet offers omega-3 fatty acids and high-quality protein from fish. Still, it risks mercury and toxin exposure, potential nutrient imbalances from over-reliance on seafood, and deficiencies if plant-based foods are neglected. Lastly, the Mediterranean diet is rich in antioxidants, healthy fats, and fiber, supporting overall health and reducing chronic diseases. However, it requires a balanced intake to avoid potential caloric excess and ensure nutrient adequacy.

**Table 2:** Summary of benefits and risks of vegetarian diets in relation to thyroid function.

Diet	Benefits	Risks	References
Vegan	Very high intake of antioxidants and anti-inflammatory compounds	High risk of iodine deficiency due to limited sources	[16]
	Supports healthy weight maintenance, reducing thyroid stress	High risk of vitamin B12 deficiency	
	Plant-based sources of selenium and zinc available (e.g., nuts, seeds)	Risk of iron deficiency due to lower bioavailability of non-heme iron	
	Promotes healthy gut microbiota due to high fiber intake	Potential risk of omega-3 deficiency without algae-based supplements	
Ovo-lacto vegetarian	High intake of antioxidants and anti-inflammatory compounds	Potential iodine deficiency, though dairy and eggs can provide some iodine	[14,16]
	Supports healthy weight maintenance, reducing thyroid stress	Potential zinc deficiency if not carefully planned	
	Adequate vitamin B12 intake from dairy products and eggs	Risk of iron deficiency due to lower bioavailability of non-heme iron	
	Eggs and dairy provide selenium, important for thyroid function		
Vegetarian	High intake of antioxidants and anti-inflammatory compounds	Higher risk of iodine deficiency compared to ovo- and lacto-vegetarians (no eggs or dairy)	[10,14]
	Supports healthy weight maintenance, reducing thyroid stress	Higher risk of vitamin B12 deficiency	
	Plant-based sources of selenium and zinc available (e.g., nuts, seeds)	Risk of iron deficiency due to lower bioavailability of non-heme iron	
	Potential benefits from fiber promoting healthy gut microbiota		
Pescatarian	High intake of omega-3 fatty acids from fish, beneficial for inflammation and heart health	Potential mercury and toxin exposure from fish consumption	[14]
	Adequate iodine intake from seafood	Risk of over-reliance on seafood for iodine and omega-3s	
	Supports healthy weight maintenance and provides high-quality protein	Some pescatarians may neglect plant-based foods, leading to fiber and other nutrient deficiencies	
	Adequate vitamin B12 intake from fish		
Mediterranean	Rich in antioxidants, anti-inflammatory compounds, and healthy fats	Potential caloric excess if not balanced	[14,18,19]
	Adequate intake of omega-3 fatty acids from fish and nuts	Potential iodine deficiency if not enough seafood is consumed	
	High in fiber, supporting gut health and weight maintenance	Need for balanced intake of diverse food groups to ensure overall nutrient adequacy	
	Provides selenium, zinc, and other essential minerals		
	Lower incidence of chronic diseases and supports overall health		

The impact of vegetarian diets on thyroid health, particularly hypothyroidism and autoimmune thyroid disorders is a multifaceted subject with varying outcomes based on dietary composition and nutrient intake. On the contrary, a vegetarian diet has been shown to have a protective effect on the development of hyperthyroidism, with flavonoids and anti-oxidants reducing the risk for autoimmune disease processes as well [19]. Research indicates both potential benefits and risks associated with vegetarianism on thyroid function, emphasizing the need for careful nutritional planning. 

Some studies suggest that a well-balanced vegetarian diet, rich in essential nutrients and low in inflammatory foods, can benefit individuals with autoimmune thyroid conditions [10,14]. This finding suggests that the anti-inflammatory properties of vegetarian diets, due to the high intake of antioxidants and phytonutrients, may help reduce autoimmune activity against the thyroid gland. The anti-inflammatory and antioxidant-rich nature of vegetarian diets can offer significant health benefits, supporting thyroid function and reducing autoimmune activity.

Conversely, other research indicates that without careful planning, vegetarian diets increase the risk of nutrient and mineral deficiencies that could worsen thyroid function [12,16,17]. Specifically, low iodine intake has been associated with an increased risk of hypothyroidism, as there is less iodine to form thyroid hormones [10,11,14]. Selenium deficiency has also been implicated in developing Grave's disease and Hashimoto thyroiditis [3,9,14]. Decreased iron stores may affect the activity of heme, thus lowering the production of thyroid hormones [15]. Zinc, vitamin D, and vitamin B12 are immunoregulatory nutrients, and their deficiencies may predispose individuals to developing autoimmune thyroid diseases [18].

Adequate intake of essential nutrients such as iodine, selenium, iron, vitamin D, and vitamin B12 is crucial for supporting thyroid and immune health in vegetarians. Careful dietary planning and possible supplementation are necessary to prevent deficiencies that could negatively impact autoimmune thyroid conditions. The risk of nutrient deficiencies poses a considerable challenge that must be addressed through strategic dietary planning and supplementation. Healthcare providers should work closely with individuals following vegetarian diets to monitor nutrient levels and recommend appropriate dietary adjustments or supplements as needed. Personalized nutrition plans that consider individual dietary preferences, lifestyle factors, and specific health needs can help optimize thyroid health while reaping the benefits of a vegetarian diet. 

The addition of meat to the diet has been a topic of interest in the exploration of its potential impact on the development of thyroid disorders, specifically hypothyroidism and hyperthyroidism. Studies have suggested that dietary factors, including meat consumption, can influence thyroid function. Some research indicates that when balanced with other food groups, moderate meat consumption may not pose significant risks to thyroid health and could even support proper thyroid function by providing necessary nutrients [14]. Meat, particularly red and processed meat, is a rich source of nutrients such as iodine, iron, and selenium, essential for thyroid hormone synthesis and metabolism [18]. However, excessive intake of these nutrients, especially iodine, can disrupt normal thyroid function, leading to hyperthyroidism [20].

Additionally, the high content of saturated fats and cholesterol in meat may trigger or exacerbate autoimmune thyroid conditions [16,21]. One study found a positive correlation between animal fat content in an individual's diet and the levels of anti-TPO or anti-Tg antibodies, likely due to increased saturated fatty acids, inducing the inflammatory response and increasing pro-inflammatory cytokines [16]. Moreover, meat from animals raised with hormones or fed with goitrogenic substances may introduce compounds that disrupt thyroid function [14,20]. Therefore, while moderate meat consumption has the potential to support thyroid health through essential nutrient provision, it is crucial to consider the type and quality of meat and the overall dietary pattern to mitigate potential negative impacts on thyroid function. 

The available studies on vegetarian diets and thyroid health, particularly concerning hypothyroidism and autoimmune thyroid disorders, are limited in number and often vary in their methodologies and participant demographics. Many studies rely on self-reported dietary intake, which can be subject to recall bias and inaccuracies. Furthermore, the long-term effects of vegetarian diets on thyroid function are not well-documented, necessitating more extensive, longitudinal research to understand these relationships better. Lastly, individual variations in nutrient absorption and metabolic responses were not extensively explored, which could influence the generalizability of the findings.

While vegetarian diets provide numerous health benefits and potentially support thyroid function, they require meticulous planning to prevent deficiencies that could negatively impact thyroid health. Ongoing research and a personalized approach to nutrition are crucial to fully understanding and harnessing the vegetarian diet's potential in managing thyroid disorders.

## Conclusions

Vegetarian diets are increasingly becoming popular globally with the goal of prioritizing a healthy, nutritious meal and preventing animal cruelty. While the benefits of a vegetarian diet have been well discussed, there is an important caveat to the detriments of this diet including that on thyroid health. Deficiencies in minerals such as selenium, iron, and Vitamin A can exacerbate the effects of iodine deficiency, leading to thyroid dysfunction. Although there are studies that suggest the anti-inflammatory effects via anti-oxidants in a vegetarian diet may have a protective effect against autoimmune disorders, there is growing evidence of significant mineral deficiencies that impact the incidence of hyper- and hypothyroidism negatively. This suggests that larger and more longitudinal studies are required to fully understand the effects of a vegetarian diet on thyroid health. 
